# Neurobiological Aspects of Mindfulness in Pain Autoregulation: Unexpected Results from a Randomized-Controlled Trial and Possible Implications for Meditation Research

**DOI:** 10.3389/fnhum.2016.00674

**Published:** 2017-01-26

**Authors:** Tobias Esch, Jeremy Winkler, Volker Auwärter, Heike Gnann, Roman Huber, Stefan Schmidt

**Affiliations:** ^1^Division of Integrative Health Promotion, Coburg University of Applied SciencesCoburg, Germany; ^2^School of Medicine, Faculty of Health, Witten/Herdecke UniversityWitten, Germany; ^3^Institute for General Medicine, University Hospital Essen, University of Duisburg-EssenEssen, Germany; ^4^Department of Psychosomatic Medicine, Medical Center, Medical Faculty, University of FreiburgFreiburg, Germany; ^5^Institute of Forensic Medicine, Forensic Toxicology, Medical Center, Medical Faculty, University of FreiburgFreiburg, Germany; ^6^Center for Complementary Medicine, Medical Center, Medical Faculty, University of FreiburgFreiburg, Germany; ^7^Institute for Transcultural Health Studies, European University ViadrinaFrankfurt (Oder), Germany

**Keywords:** meditation, mindfulness, pain tolerance, attention, morphine, opioids, placebo

## Abstract

**Background:** Research has demonstrated that short meditation training may yield higher pain tolerance in acute experimental pain. Our study aimed at examining underlying mechanisms of this alleged effect. In addition, placebo research has shown that higher pain tolerance is mediated via endogenous neuromodulators: experimental inhibition of opioid receptors by naloxone antagonized this effect. We performed a trial to discern possible placebo from meditation-specific effects on pain tolerance and attention.

**Objectives:** It was proposed that (i) meditation training will increase pain tolerance; (ii) naloxone will inhibit this effect; (iii) increased pain tolerance will correlate with improved attention performance and mindfulness.

**Methods:** Randomized-controlled, partly blinded trial with 31 healthy meditation-naïve adults. Pain tolerance was assessed by the tourniquet test, attention performance was measured by Attention Network Test (ANT), self-perceived mindfulness by Freiburg Mindfulness Inventory. 16 participants received a 5-day meditation training, focusing on body/breath awareness; the control group (*N* = 15) received no intervention. Measures were taken before the intervention and on 3 consecutive days after the training, with all participants receiving either no infusion, naloxone infusion, or saline infusion (blinded). Blood samples were taken in order to determine serum morphine and morphine glucuronide levels by applying liquid chromatography-tandem mass spectrometry analysis.

**Results:** The meditation group produced fewer errors in ANT. Paradoxically, increases in pain tolerance occurred in both groups (accentuated in control), and correlated with reported mindfulness. Naloxone showed a trend to decrease pain tolerance in both groups. Plasma analyses revealed sporadic morphine and/or morphine metabolite findings with no discernable pattern.

**Discussion:** Main objectives could not be verified. Since underlying study goals had not been made explicit to participants, on purpose (framing effects toward a hypothesized mindfulness-pain tolerance correlation were thus avoided, trainees had not been instructed how to ‘use’ mindfulness, regarding pain), the question remains open whether lack of meditation effects on pain tolerance was due to these intended ‘non-placebo’ conditions, cultural effects, or other confounders, or on an unsuitable paradigm.

**Conclusion:** Higher pain tolerance through meditation could not be confirmed.

## Introduction

Pain as an unpleasant sensory and emotional experience has a multidimensional nature, which means it is sensitive to diverse manipulations, implicating there are many starting points for pain relief. Keeping in mind that, for example, chronic pain patients cost about 38 billion Euros per year in Germany (Zimmermann, 2004), reliable therapeutic concepts are desperately needed. A better understanding of the notion of pain is mandatory.

### Pain Manipulation through Mindfulness and Meditation

When observing the phenomenon of pain it appears that there are several dimensions involved, from which the affective and cognitive dimension are two: the affective dimension reflecting the emotional and motivational relevance of the stimuli and the cognitive dimension relating to how aspects of cognition can sculpt one’s experience (Melzack and Casey, 1968). It seems quite logical that manipulation on these two dimensions would lead to an altered pain experience. Meditation, understood as a mental training that shapes the brain/mind in highly specific ways, is a relatively new approach in the field of pain modulation (Benson et al., 1974; Hoffman et al., 1982; Kabat-Zinn, 1982, 2003; Kabat-Zinn et al., 1985; Lazar et al., 2000; Esch et al., 2003; Salamon et al., 2006; Zeidan et al., 2010, 2011, 2012; Schmidt et al., 2011) and could be one of such starting points among others to manipulate the affective and cognitive dimensions of pain.

Meditation lies at the core of mindfulness-based stress reduction (MBSR). MBSR, since its development for chronic pain and stress-associated disorders in the late 1970s, is under intensive research (Kabat-Zinn, 1982; Kabat-Zinn et al., 1985; Bishop et al., 2004; Grossman et al., 2004; Ernst et al., 2009; Zeidan et al., 2010, 2011, 2012; cf. Bazarko et al., 2013; Katterman et al., 2014), and now frequently used in clinical practice. Evidence, however, for the clinical effectiveness of MBSR programs in improving pain intensity or disability, e.g., in chronic low back pain patients, is still inconclusive, with limited evidence that MBSR can improve pain acceptance (Cramer et al., 2012). A recent randomized clinical trial by Cherkin et al. (2016) concluded that MBSR may be an effective treatment option for patients with chronic low back pain, though effects were similar to those of cognitive behavioral approaches, leaving the question open as to the specificity of effects in mindfulness training. Kabat-Zinn (2003, p. 145) defines mindfulness as “the awareness that emerges through paying attention on purpose, in the present moment, and non-judgmentally to the unfolding of experience moment by moment.” Mindfulness has been suggested to be effective via four mechanisms (Hölzel et al., 2011): attention regulation, body awareness, emotion regulation, and changes in perspective on the self.

This study was planned and conducted in light of recent evidence demonstrating, indeed, that meditation alters pain perception (e.g., reduction of subjective pain ratings in healthy adults), and that pain tolerability for experimental pain is elevated – since, for example, a short mindfulness training has been shown to increase pain tolerance in novices (Kabat-Zinn, 1982; Kabat-Zinn et al., 1985; Kingston et al., 2007; Zeidan et al., 2010, 2011, 2012; MacCoon et al., 2012); however, some of these early experimental studies on pain modulation through meditation training didn’t involve proper controls or randomization procedures. The postulated phenomenon has recently been called ‘meditative analgesia’ by some authors (Grant, 2014), also discussing various or ‘specific’ forms of meditation that are particularly linked to the presumed potential of affecting pain.

In addition, there is evidence for a modification of the subjective interpretation of pain via meditation in patients dealing with chronic pain (cf. Schmidt et al., 2011). However, little is known about underlying neurobiological mechanisms. For example, the involvement of dopamine or dopaminergic brain pathways in meditation (Bujatti and Biederer, 1976; Kjaer et al., 2002), and the production of endogenous opioids/opiates via dopamine (Zhu et al., 2005; Kream and Stefano, 2006; Stefano and Kream, 2007, 2010; Stefano et al., 2007, 2012; Mantione et al., 2008; Zhu and Stefano, 2009; Esch, 2014), have been discussed. An activation of these pathways could, theoretically, influence pain modulation. Also, it has been speculated that pain attenuation through mindfulness might involve unique brain mechanisms that are in sharp contrast to established models, e.g., comprising increased sensory processing and decreased cognitive control (Gard et al., 2011). Accordingly, a search for modulators and key neurotransmitters, or autoregulatory indicators, of this proposed shift has begun.

### Neurobiological Background and Hypotheses Generation

In this regard, endogenous opioidergic (morphinergic) pathways and agonistic signaling on mu3 and mu4 opioid receptors in the brain received attention in meditation research, as well as a coupling of related dopaminergic and nitric oxidergic pathways, converging or originating in brain regions that critically process pain (Bujatti and Biederer, 1976; Dusek et al., 2005; Mantione et al., 2007; Jung et al., 2010; Hölzel et al., 2011; Kemper et al., 2015). Part of the research background is that the potential of endogenous production of morphine via dopamine has already been shown, and related molecular pathways have been identified (Zhu et al., 2005; cf. Kream and Stefano, 2006; Stefano and Kream, 2007; Stefano et al., 2007, 2012; Mantione et al., 2008; Zhu and Stefano, 2009); specific binding of morphine to the mu (mu3/mu4) opioid receptor has been demonstrated (Guarna et al., 2003; Cadet et al., 2004, 2007; Stefano et al., 2004, 2008a; Zhu et al., 2004a; Mantione et al., 2006, 2010; Kream et al., 2007; Welters et al., 2007; Fricchione et al., 2008; Ghelardini et al., 2008; Zhu and Stefano, 2009; Pasternak and Pan, 2013). In addition, morphine activates nitric oxide (NO)-producing enzymes, and the activation of constitutive NO-synthases via morphine has been demonstrated (Tseng et al., 2000; Welters et al., 2000; Mantione et al., 2003, 2006, 2008; Stefano et al., 2004, 2005, 2008b, 2009; Zhu et al., 2004a,b; Casares et al., 2005; Pak et al., 2005; Cadet et al., 2007; Kream and Stefano, 2009, 2010; cf. Banach et al., 2010). In fact, coupling of constitutive NO to cellular/physiological stress and pain reduction has been discussed for long (cf. Esch et al., 2002). Finally, there exists clear evidence for an elevation of pain tolerance via opiate alkaloids (such as morphine) or opioid peptides – be it externally administered or internally/endogenously activated (e.g., Amanzio and Benedetti, 1999; Hutchinson et al., 2004; Stefano et al., 2009; Bodnar, 2013; Hajj et al., 2013; Miguez et al., 2014). Thus, we surmised that opioidergic/morphinergic mechanisms might play a role in assumed meditation-related alteration of pain tolerability.

### Possible Placebo Analogies

Interestingly, quite similar mechanisms have already been demonstrated for the placebo effect and related pain-associated phenomena, and their physiology in this regard. This includes evidence for the involvement of dopamine in placebo response (de la Fuente-Fernández et al., 2001, 2006; Stefano et al., 2005; Scott et al., 2008; de la Fuente-Fernández, 2009; Lidstone et al., 2010), or an involvement of opioid transmission, i.e., opioid signaling mechanisms and/or receptors (Benedetti and Amanzio, 1997; Amanzio and Benedetti, 1999; Benedetti et al., 1999a,b, 2005, 2006; Amanzio et al., 2001; Ribeiro et al., 2005; Zubieta et al., 2005; Scott et al., 2008; Schoell et al., 2010). Furthermore, central mu opioid receptor (sub-) systems seem to play a significant role in the placebo response, as do limbic system (e.g., nucleus accumbens, anterior cingulate), prefrontal (e.g., dorsolateral prefrontal cortex) and insular cortices (among others: e.g., amygdala, periaqueductal gray matter), presumably also via imbedded mu opioid receptor activation (cf. Ribeiro et al., 2005; Zubieta et al., 2005).

Evidence exists for the involvement of the mu opioid receptor system in placebo-analgesia (e.g., Ribeiro et al., 2005; Zubieta et al., 2005; Schoell et al., 2010), and for an increment (elevation) of pain tolerance by active elicitation of the placebo response (e.g., Amanzio and Benedetti, 1999). Finally, the opioid antagonist naloxone has been shown to reduce pain tolerance in placebo studies on experimental pain tolerability (e.g., Amanzio and Benedetti, 1999; Schoell et al., 2010), or conditioning experiments (e.g., Flor et al., 2002), again showing that effects are processed, at least partially, via the endogenous opioidergic system. In fact, there is evidence that naloxone selectively binds to mu opioid receptors, i.e., it particularly antagonizes mu opiate signaling at least at lower or ‘physiological’ concentrations; some authors calculate naloxone affinity for the mu receptor, as compared to delta or kappa opioid receptors, with 20:1 (e.g., Zadina et al., 1993; Chong et al., 2006; Kim and Richardson, 2009; Tsai et al., 2009; Jan et al., 2011). Importantly, naloxone itself has no pain modifying effects, i.e., it does not alter pain tolerance *per se* (Grevert and Goldstein, 1978; Amanzio and Benedetti, 1999). Given this, the reduction of pain tolerance following naloxone administration in placebo studies (incorporating experimental pain models), as demonstrated, may be linked to opioidergic, that is: morphinergic, mu receptor signaling.

### Study Objectives

On the basis of these considerations, we decided to study the neurobiological aspects of pain modulation through mindfulness-based meditation techniques in healthy participants.

We expected an increased pain tolerance in the meditation training group. Comparable to the placebo effect, we speculated that pain modulation is mediated via endogenous, opioidergic mechanisms. Related opioid/opiate compounds (e.g., morphine and its metabolites) should therefore be found in the plasma of blood samples of the study participants, and also be blocked by administration of opioid antagonists, such as naloxone. This effect would imply an involvement of the mu opioid receptor, which is particularly sensitive to naloxone and which has – with its subtypes mu3 and mu4 – a high affinity to the opiate alkaloid morphine.

Hence, the hypotheses (objectives) of our study were as follows:

(A) Meditation increases pain tolerance in healthy adults (pre meditation training compared to post, and intervention group compared to non-meditating control group);
(B1) Effects of meditation on pain modulation/perception are mediated via opioid mechanisms and can therefore be blocked by administration of the opioid antagonist naloxone;
(B2) Endogenous morphine is involved in meditation-dependent pain modulation and can therefore be detected in the plasma of study participants (blood collected pre and post; plasma analyzed by liquid chromatography-tandem mass spectrometry for morphine and morphine glucuronides – M3G, M6G);
(C) Increased pain tolerance following meditation training (see A) correlates with improved attention performance [as measured by the Attention Network Test (ANT)], as well as increased self-perceived mindfulness [assessed by the Freiburg Mindfulness Inventory (FMI)].

## Materials and Methods

### Design

This study was conducted as a randomized control trial (RCT). Procedures were conducted in a partly blinded manner (see below). The trial lasted 10 days with five measurement points. Two assessments took place before the intervention (days 1 and 2) followed by the intervention taking place on five consecutive days (days 3–7) and three assessments after the intervention (days 8–10).

### Participants

The study sample consisted of participants who were recruited via informative postings throughout university campus (Freiburg University), as well as announcements on an internal digital message board for employees. Participants had to be at least 18 years old, language proficient, and without visual impairment that would prevent them from completing the research assessments. Excluded were those with any form of addiction, regular use of pain medication, severe psychiatric disease, epilepsy, diabetes, pregnancy or prior meditation experience. Participation included five visits to the University Medical Center for assessment lasting approximately 2 h (days 1, 2, 8–10) and for participants randomized to the invention group five meditation sessions (1.5 h, days 3–7). Participation was voluntary, and all participants received a compensation of 150 €; for their participation. We calculated a sample size of ≥30 which was in accordance with prior studies on that topic and relevant standard protocols (cf. Benedetti et al., 1999a; Zeidan et al., 2010, 2011).

All participants gave written informed consent.

### Intervention

The participants were randomized either to a passive control condition (no intervention *N* = 15) or trained in a combined breathing/mindfulness meditation technique (intervention group *N* = 16) for five consecutive days – i.e., five daily group sessions of 1.5 h each. The topic of pain (e.g., pain awareness or pain perception) was intentionally and carefully avoided in this course. Training took place every day from 11:30 am until 1:00 pm.

The trainer (TE) had 20 years of meditation experience, and is a professional meditation/mindfulness teacher, and researcher in the field. Each training session consisted of feedback rounds and exchanges on personal experiences, followed by 20–25 min of formal group mindfulness meditation practice (techniques taught: body scan, attention to breath (ATB), attention to senses (ATS), open awareness/attention to experience (ATE), and walking meditation – with focused breath awareness as a steady anchor), and a length of 40 min on the last day. Another feedback round followed each daily meditation practice before the session closed. Participants were also motivated to add another short/informal meditation practice during the day/evening, left to their personal preference and choice (e.g., “mini meditation” – only a few breaths a couple of times during the day – or another maximum 10–20 min of formal practice). This suggestion was meant to individually complement daily group meditation sessions, echoing and possibly deepening the training. However, this suggested additional practice was not formally assessed.

### Measures

#### Pain Tolerance

Pain tolerance was measured two times before and three times after the training in each participant through ischemia induction in the forearm following a standard protocol (Benedetti et al., 1999a), referred to as tourniquet test. In their original experiments (Amanzio and Benedetti, 1999) investigated the mechanisms underlying the activation of endogenous opioids in placebo analgesia by using the model of human experimental ischemic arm pain. Different types of placebo analgesic responses were evoked by means of expectation, conditioning, or a combination of both.

In our experiments, following this experimental setup, participants reclined on a bed and 10 ml of blood was drained from their dominant forearm. Next they had to extend their non-dominant arm vertically and an Esmarch bandage was placed. The bandage was used to squeeze the blood out of the arm. A sphygmomanometer cuff was placed around the upper arm and inflated to a pressure of 300 mm Hg to keep the arm virtually empty of arterial blood supply, and a stopwatch was started. After this, the bandage was removed and the arm lowered on the participant’s side. Then, the participant had to squeeze a hand exerciser 12 times by pressing the exerciser for 2 s and then resting for 2 s. The force necessary to bring the handles together was 6.5 kg. The ischemic pain induced by this procedure increased over time. Participants were asked to tolerate the pain until they get the impression of not being able to withstand it any longer. Once the participant indicated that this point was reached the stop-watch was stopped and the cuff was immediately deflated. Three minutes after the end of the tourniquet test a second blood sample of 10 ml was taken from the dominant forearm.

On two of the three post-intervention assessments on days 9 and 10, immediately before the pain tolerance measurements, participants received a blinded infusion of either naloxone or saline on either day 9 or day 10 in randomized sequence. Following a blinded, randomized protocol the infusion was administered by a perfusor via a venous port. Naloxone concentration was 0.14 mg/kg in 0.9% NaCl-solution with 0.1 ml/s; the total infusion time was 180–250 s. The infusion port was kept open until 8 ml of NaCl-solution passed. Study assistants as well as participants were blinded to the content of the infusion. As explanation for the i.v. line participants were told that a neutral substance without any effect was administered, usually used for detoxification and now applied in this study to facilitate the assessment of certain blood parameters. The procedures followed a standard protocol (see Amanzio and Benedetti, 1999). After day 10, all participants had received hidden naloxone and saline, once in each case.

#### Morphine and Morphine Metabolites

**FIGURE 1:**
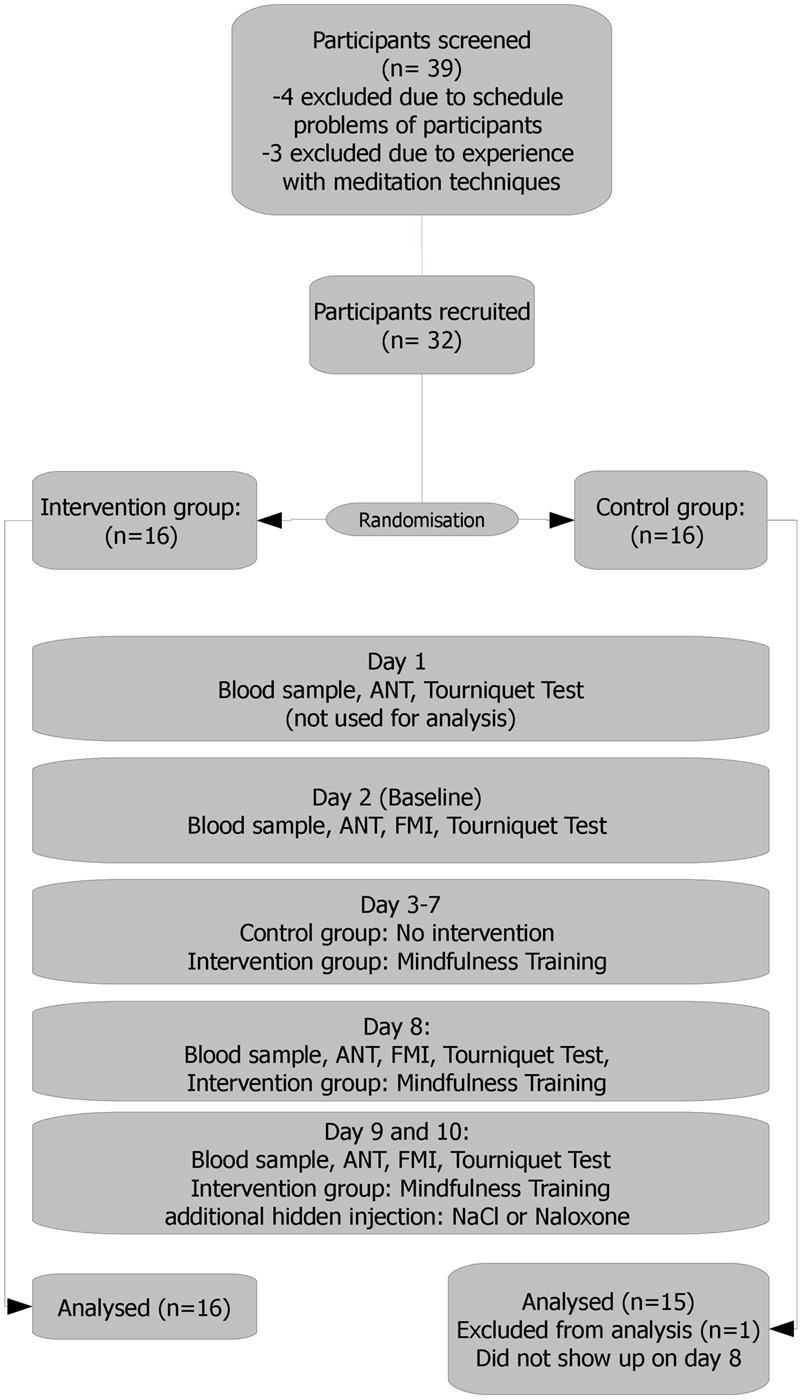
**Study flow.** Day 1: a personal talk with each participant, scale weight. Results held no entrance in the analysis and served only as a test run, making the participants familiar (acquainted) with the setting. Day 2: results served as the baseline. Days 3–7: mindfulness training/no intervention Day 8: intervention group: 10 min meditation practice before experiment. Days 9 and 10: 10 min meditation practice for the intervention group. Naloxone or saline administration. Naloxone or saline were administered via a venous port following a blinded, randomized and controlled protocol.

Total morphine concentrations after enzymatic cleavage of glucuronides and the concentrations of the morphine metabolites morphine-3-glucoronide (M3G) and morphine-6-glucoronide (M6G) were measured in the participants’ plasma samples collected during the study by liquid chromatography-tandem mass spectrometry. From each participant 10 blood samples were collected pre and post-training (see **Figure 1**). Serum was separated from the blood clot after centrifugation and stored at -20°C until analysis. Each sample was measured after enzymatic cleavage using β-glucuronidase (*Escherichia coli*, 140 U/mg, 1 h at 37°C), and without enzymatic cleavage. After cleavage (1.5 ml serum, 0.5 ng D_3_-morphine as internal standard, 2 ml phosphate buffer pH 6, 50 μl β-glucuronidase) solid phase extraction (SPE) was performed using mixed mode cation exchange cartridges (CHROMABOND Drug, 200 mg, Macherey-Nagel, Düren, Germany) on an automated SPE device (GX-274 ASPEC, Gilson, Middleton, WI, USA). For the measurement without enzymatic cleavage 2 ml serum was mixed with 2 ml of phosphoric acid (4%) and internal standard (0.5 ng D_3_-morphine, D_3_-M3G, and D_3_-M6G each). For SPE Oasis^®^ MCX cartridges (60 mg, Waters, Milford, MA, USA) were used on a GX-274 ASPEC (Gilson, Middleton, WI, USA). Chromatographic separation was performed on a Shimadzu 1100 Series HPLC system equipped with a Kinetex column (100 mm × 2.1 mm, 2.6 μm particle size; Phenomenex, Aschaffenburg, Germany) applying gradient elution. A QTrap 5000 triple quadrupole linear ion trap mass spectrometer fitted with a TurbolonSpray interface (Applied Biosystems/Sciex, Darmstadt, Germany) was used in MRM mode (positive mode) for detection of the target compounds. Two ion transitions were monitored per analyte and for quantitation the peak area ratios of the target compound to the analogous deuterated internal standard (D_3_-morphine, D_3_-morphine-3-glucuronide, and D_3_-morphine-6-glucuronide) were used. Lower limit of quantitation (LLOQ) was 2 pg/mL for morphine and 25 pg/mL for both morphine glucuronides. Calibration was linear from 2 to 15 pg/mL for morphine and from 25 to 200 pg/mL for the morphine glucuronides.

#### Attentional Network Performance

We measured the attention performance with the ANT (Fan et al., 2002), which we also used as a control to see whether or not the meditation training was effective. The ANT test assesses in parallel three different components of the attentional network, i.e., the alerting, the orienting, and the executive component. The basic task of the ANT is a flanker task which is either facilitated or impeded by the application of cues and distractors. The dependent measure is reaction time (RT). Overall, six different conditions are presented resulting from three cue conditions (no cue, center cue, spatial cue) and two target conditions (congruent and incongruent). Performance indicators for the three attentional components are obtained by computing several RT differences between these six conditions. The ANT test was performed on day 2 (baseline) and days 8–10 before the tourniquet-test. The test consisted of six blocks (of 48 trials with no feedback which were distributed over the assessment days). About 3 min of practice procedure was held before the experiment.

#### Self-Reported Mindfulness

##### Freiburg Mindfulness Inventory

The FMI (Buchheld et al., 2001; Walach et al., 2006) is a short form with 14 items suitable also for participants without profound background knowledge in mindfulness. It provides a reliable and consistent scale evaluating important aspects of mindfulness. It is considered as one dimensional for practical purposes. Items are scored on a four point Likert scale ranging from 1 (strongly disagree) to 4 (strongly agree). Participants filled in the questionnaire on day 2 (baseline) and days 8–10 before the tourniquet-test.

### Procedures

Possible participants for the study were pre-screened via phone calls, following initial contact through postings, etc. If they fulfilled inclusion criteria they were sent information documents and a date for their assessment. During initial assessment, eligibility was cross-checked and basic instructions provided. For actual study enrolment, participants were distributed among verum (intervention) and control group following a standard randomization protocol. Upon successful enrolment, each participant received an anonymized identification number for further procedures. Enrolment stopped when calculated sample sizes were met for both groups. Group assignment took place by randomizing the whole sample (*N* = 32) at once into two groups of equal size and was performed by a statistician who was not involved in participant interaction. Group assignment was noted in opaque and sealed envelopes with the identification number on top of the envelope. Experimenters and study nurses performing tourniquet tests, ANT tests and blood samples were kept blind to the participants’ group assignment. Participants were informed about their individual group assignment (to get to know whether they would be required to show-up for intervention training) after the completion of assessments on day 2 by a person otherwise not interacting with the participants. Participants were told not to speak with other participants about group assignment. For additional assignments – regarding the sequence of hidden saline/naloxone administration on days 9 and 10 – both groups (*N* = 16, each) were randomized separately again in one step in order to get four groups of the same size (*N* = 8). Group assignment was again noted in sealed opaque envelopes. Similarly to experimenters/study nurses, participants were kept blind about these additional assignments. Naloxone and NaCl infusions were prepared by two anesthesia nurses in special laboratory in a different part of the building directly before the application. The nurses opened the envelope and prepared the infusions accordingly which were then handed over to the MDs. The nurses had no direct interaction with the participants.

All participants were required, and instructed as such, to strictly avoid poppy/poppy seed consumption during study enrolment and the course of the study to avoid positive morphine or morphine glucuronide findings caused by adhering alkaloid residues (from external sources). Overall duration of the study was 10 days, following an adjusted standard protocol (Amanzio and Benedetti, 1999) (see also flow chart – **Figure 1**):

*Day 1:* A personal introduction was given to each participant, explaining the procedures in-depth and discussing remaining questions. Participants then gave informed consent and filled in questionnaires (FMI, sociodemographics). After this, each participant was weighed. Then the first run of the ANT and pain tolerance test (tourniquet) was conducted. Results held no entrance in the analysis and served only as a test run, making the participants familiar with the setting.
*Day 2:* Attention Network Test, FMI, and tourniquet test were measured. Results served as baseline.
*Days 3–7:* Depending on the group assignment, participants were trained in the described meditation techniques (for 90 min) or had no task.
*Day 8:* Participants who were randomized in the intervention group had their meditation practice (10” short group meditation – breath awareness). ANT, FMI, and tourniquet test were conducted for all participants thereafter.
*Days 9 and 10:* Meditation practice (10” short group meditation – breath awareness) for the intervention group. After that ANT, FMI and tourniquet test were conducted, this time with either hidden naloxone or saline administration.

### Sample Size, Data Analysis, and Statistics

Sample size consideration was based on a power analysis with an estimated effect size of *d* = 0.92 (α = 0.05, 1-β = 0.80) and resulted in *N* = 32 (both groups). The power analysis was based on the protocol and findings of Amanzio and Benedetti (1999).

Baseline measurements were compared by *t*-test for independent measures or *Chi^2^* test for differences. Hypothesis A was assessed by an ANCOVA taking the baseline data as covariates and this procedure was also applied to assess post-intervention changes for the various indicators of the ANT as well as for the FMI scores. Hypothesis B1 was tested by a repeated measurement ANOVA for the naloxone and saline assessment with baseline data (day 2) taken up as covariate. Here, we had the factors *group* (between-subjects, intervention versus control) and *infusion* (within-subject, naloxone versus saline). For this hypothesis the *group × infusion* interaction was the relevant indicator. Effect sizes were either partial η^2^ from the respective analysis or Cohen’s d computed as the differences of the means divided by the pooled standard deviation. Correlations were computed with Spearman’s rho. All analyses were conducted by SPSS 21.

## Results

### Participants

Thirty-two participants were included and showed up on day 1. One participant of the control group did not show up on days 8, 9, and 10 and had to be excluded. Groups are matching in age and weight. Basic sample characteristics are depicted in **Table 1**.

**Table 1:** Sociodemographic data of the intervention and control group (SD, standard deviation).

	Intervention group	Control group	*p*
*N*	16	15	
Mean age (SD)	27.8 (8.41)	25.5 (6.79)	0.42
Sex (m/f, absolute numbers)	5/11	3/13	0.41
Mean weight in kg (SD)	62.6 (12.70)	58.5 (11.43)	0.35

### Pain Tolerance

The main outcome was time to stand an ischemic pain stimulus (tourniquet test, see **Table 2**). Pre-intervention baseline was 12 min 58 s for the intervention group and 13 min 18 s for the control group. On the first post-intervention measurement (day 8), intervention group gained 2 min 01 s (14 min 59 s, *d* = 0.27), control group gained 5 min 37 s (18 min 53 s, *d* = 0.54). The difference (Hypothesis A) was not significant (ANCOVA, *F* = 2.28, *df* = 1/28, *p* = 0.14, $\eta_{p}^{2}$ = 0.075). The pain test was repeated on days 9 and 10, immediately after receiving either isotonic NaCl (saline) or naloxone via a venous port. In the intervention group, pain tolerance under naloxone remained technically unchanged (it was extended by 0 min 06 s, to 13 min 04 s) and was extended by 1 min 04 s under saline (placebo), compared to baseline. The control group showed an extension of 3 min 00 s (16 min 18 s) for naloxone and 3 min 42 s (17 min 00 s) for placebo. Taken both groups together participants could tolerate pain longer by 50 s under placebo (15 min 28 s, *SD* = 6 min 32 s) than under naloxone (14 min 38 s, *SD* = 6 min 47 s); this difference was not significant (*t*-test for dependent means, *T* = 1.46, *df* = 30, *p* = 0.16, *d* = 0.13). There was no significant difference between groups for the difference between naloxone and placebo (Hypothesis B1, repeated measurement ANOVA *F* = 0.52, *df* = 1/28, *p* = 0.82, $\eta_{p}^{2}$ = 0.002).

**Table 2:** Results of tourniquet test, mean time to stand ischemic pain in min:sec (SD) in the intervention group and control group.

Tourniquet test	Day2 Baseline	Day 8	Day 9	Day 10	Naloxon	Placebo
Intervention group	12:58 (6:00)	14:59 (8:29)	13:13 (6:14)	13:53 (7:33)	13:04 (7:01)	14:02 (6:49)
Control group	13:18 (7:27)	18:53 (12:26)	16:50 (6:17)	16:29 (6:08)	16:18 (6:20)	17:00 (6:04)

### Morphine

Mass spectrometric plasma analyses for morphine and morphine glucuronides (M3G and M6G) revealed sporadic measures in the pg/ml range (morphine: 2.8–221 pg/ml [LOQ: 2 pg/ml]; M3G: <25–417 pg/ml [LOQ: 25 pg/ml]; M6G: <25–35 pg/ml [LOQ: 25 pg/ml]). Regarding correlations between morphine detection and pain tolerance on intraindividual levels (i.e., within subject), our analyses revealed higher pain tolerance with morphine co-occurring in seven out of nine cases (as compared to series of pain measures in these subjects without proof of morphine in the plasma). When morphine metabolites (M3G, M6G) were additionally present in the plasma, that is, coincidentally with morphine, six out of seven subjects showed higher pain tolerance. Given the inability to confirm our initial study objective (i.e., discrimination of pain tolerance between groups due to mindfulness training/meditation intervention), we abstained from more detailed elaboration on individual/sample levels, or case studies on possible associations between individual morphine values, naloxone effects, pain tolerances, and mindfulness.

With no discernable patterns at general levels, and only incidental opiate measures (36 positives in 310 samples, with 12 samples showing very low morphine concentrations only with glucuronides not detected), we could only speculate whether morphine values originated form external, alimentary sources, or referred to its actual endogenous release. Possibly, morphine bioavailability as well as first-pass effect (e.g., liver metabolism) would appear to make exogenous sources less probable. However, we conducted additional experiments involving mass spectrometric plasma analyses (not depicted here), trying to describe and further narrow down ranges for possible alimentary sources (e.g., accidental poppy seed consumption). The results showed that morphine, M3G and M6G concentrations reached in the study samples could be explained by unintentional intake of poppy seed-containing food, since even small amounts of poppy seeds, which may not be noticed, e.g., in bakery products, can be sufficient to produce positive blood morphine and morphine glucuronide findings in the range of concentrations detected within the study population. Taken together, despite instructing the participants to abstain from consumption of poppy/poppy seeds, external sources for morphine and metabolite detection in the study samples cannot be ruled out completely. Results have thus to be dealt with great care, which is why we refrained from further analysis and interpretation.

### Attention

Attention performance was tested with ANT: data for RT and error scores were analyzed separately.

### Reaction Time

Overall, participants improved significantly in RT at post-intervention assessment (471 ms compared to 496 ms, *T* = 4.407, *df* = 30, *p* < 0.001, *d* = 0.39). We computed ANCOVAS for the three ANT indicators as well as for the overall RT and none of these variables showed significant group differences (see **Table 3**).

**Table 3:** Attention Network Test (ANT) results for reaction time differences (ms). Differences between groups at post-test based on ANCOVA with pre-test as covariate.

		Intervention group		Controls					
		*Mean*	*SD*	*Mean*	*SD*	*F*	*df*	*p*	$\eta_{p}^{2}$
Overall	Pre	499	76.7	493	81.8	0.141	1/28	0.71	0.005
	Post	474	60.9	467	47.4				
Alerting	Pre	33.66	21.006	31.47	14.760	0.646	1/28	0.43	0.023
	Post	40.45	20.832	34.07	16.195				
Orienting	Pre	53.10	17.644	46.77	20.778	3.033	1/28	0.09	0.098
	Post	41.30	19.553	42.83	20.231				
Executive	Pre	70.75	25.637	76.02	47.104	0.007	1/28	0.93	0.000
	Post	59.61	21.239	61.64	23.326				

#### Error Score

**FIGURE 2:**
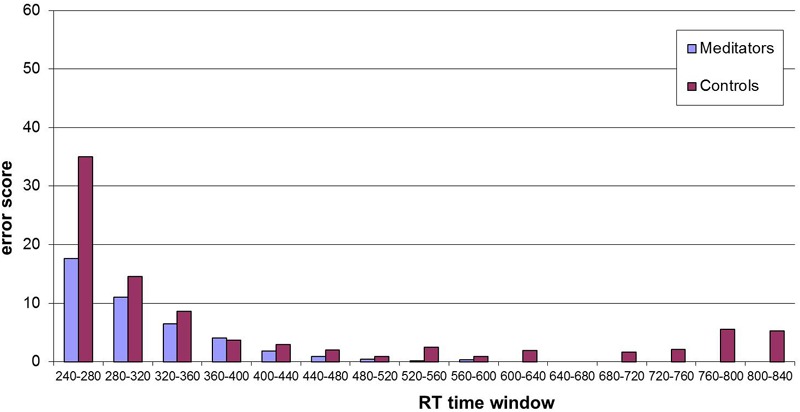
**Histogram of error scores at post-intervention for different reaction time (RT) windows (15 bins, width 40 ms).** Distribution analysis showed significant differences between the two groups (*z* = 3.23, *p* = 0.001 – see text).

The error score describes the amount of errors participants made (i.e., pressing the wrong button) while performing the task. Since there is always a speed-accuracy trade-off, changes are interesting with respect to the shorter RT found after the intervention. Overall, participants made less error after the intervention at the second assessment (2.76 compared to 2.65), but this reduction was not significant (*T* = 0.374, *df* = 30, *p* = 0.71). If analyzed for group differences, the groups showed a tendency to perform different, with the meditators reducing error after the intervention (see **Table 4**). If these error rates are split up with respect to different RT as showed in the histogram (**Figure 2**) one can see that meditators showed especially a better performance in very short RTs. We compared the distribution of meditators and controls with the non-parametric Wilcoxon Test and found a significant difference (*z* = 3.23, *p* = 0.001).

**Table 4:** Attention Network Test results for error score.

		Intervention group		Controls					
		*Mean*	*SD*	*Mean*	*SD*	*F*	*df*	*p*	$\eta_{p}^{2}$
Overall	Pre	2.51	2.787	3.02	2.200	3.887	1/28	0.059	0.122
	Post	1.98	1.750	3.35	2.601				

### Self-Attributed Mindfulness (FMI)

We assessed self-attributed mindfulness at baseline and after the intervention. Data are displayed in **Table 5**. Here, larger values indicate a higher level of self-attributed mindfulness. No differences were found between the groups at baseline (*T* = 0.471, *df* = 29, *p* = 0.64) and after the intervention (see **Table 5**).

**Table 5:** Results of Freiburg Mindfulness Inventory.

		Intervention group		Controls					
		*Mean*	*SD*	*Mean*	*SD*	*F*	*df*	*p*	$\eta_{p}^{2}$
FMI	Pre	37.0	4.50	37.9	6.42	0.026	1/28	0.87	0.001
	Post	37.9	2.98	38.3	6.32				

*Test-statistic for differences at post-intervention (ANCOVA) with pre intervention values taken as covariate.*

### Correlational Analyses

We correlated change scores of FMI and ANT (Alerting, Orienting, Executive, Error Score) with improvement in pain from day 2 to day 8 for the meditation group only. None of these correlations turned out significant. However, there was a medium size correlation between increase of self-attributed mindfulness and increase in pain tolerance (Spearman’s ρ = 0.33, n.s.). In the whole sample this correlation reached significance with 0.36 (*p* = 0.046).

## Discussion

With our study, we wanted to learn more about possible mechanisms in pain regulation and the neurobiology behind alleged pain-reducing effects of mindfulness meditation (i.e., short novices training). Therefore, we applied established protocols for experimental pain, and meditation training, also considering an overlap in physiology between placebo and meditation autoregulation.

Claiming rigorous methodology, our study involved partial blinding of participants and examiners, as well as randomization and controlling procedures. In addition, the topic of pain, its reduction or increased tolerance, was carefully avoided during meditation training to eliminate vectored expectations. We used standard procedures for experimental pain stimulation from other placebo models (established placebo experiments). Our hypotheses were that meditation training would lead to an increased experimental pain tolerance, naloxone could inhibit this effect, and an increased pain tolerance would, presumably, correlate with improved attention performance, and mindfulness.

As described, we were unable to discern and yet confirm an increase of pain tolerance exclusively for the intervention group (i.e., meditation training).

An indicator that our meditation training had an influence on the participants can be drawn from the results from the ANT. While there was no change in the RT for the three ANT indicators, meditators showed a significantly different error score distribution (post-training). This result is in accordance with other meditation studies, using the ANT (Tang et al., 2007; van den Hurk et al., 2010; Jo et al., 2016), where the same pattern, i.e., fewer errors in trials with short RT following meditation training, was found: the ANT results showed a statistically significant smaller error score, especially for fast RTs. This is also consistent with earlier findings that showed improvements in the efficient distribution of attentional resources among competing stimuli for meditation trained subjects (Kerr et al., 2011). We furthermore found a significant correlation between changes in self-attributed mindfulness (FMI) and changes in pain tolerance for the entire sample of both groups combined. This correlation might be due to repeated testing, but if not it would indicate that self-attributions regarding being present and acceptant are somehow related with pain tolerance. Taken into account the fact that FMI results, surprisingly, did not differ between groups the presence of this correlation in both groups is consistent.

Increases in pain tolerance that were, indeed, measurable occurred in both groups. They were particularly dominant in the control group, which would, counterintuitively, reject our initial hypothesis. Again, this could represent an effect of learning, i.e., getting used to the experimental procedures over time, although still hypothetical. We are aware of one study that explicitly covers the topic of habituation from repeated representation of painful stimuli of fixed intensity by assessing the effect of mindful attention on pain habituation (Ginzburg et al., 2015). However, we were unable to identify additional studies confirming such supposable development of tolerance with repeated exposures to pain test stimuli. The incidental or structural nature of our findings thus remains an open question at this point.

Naloxone showed a trend to decrease pain tolerance in both groups, but plasma analyses for morphine and morphine compounds (metabolites) revealed only sporadic measures, with no clear or discernable patterns at this level. Accordingly, a possible impact, or significance, of opioidergic/morphinergic signaling in meditation and pain physiology remains largely unanswered at this point. Interestingly, two recent studies found contradictory evidence in this regard: Zeidan et al. (2016), conducted a study on meditation-based pain relief, also using naloxone as a possible antidote. However, they failed to reverse meditation-induced analgesia by this and yet concluded pain-reducing effects not to be mediated by endogenous opioids. In sharp contrast, Sharon et al. (2016) found that mindfulness meditation-induced analgesia in their trials was reversed by naloxone in an experimental pain model also using the presentation of noxious temperature as pain stimulus (Sharon used cold; Zeidan used heat). They additionally described meditation effects matching placebo physiology.

### Neurobiological Speculations: Where to Go from Here?

We won’t decide at this point which direction future evidence will take. Certainly, optimum “dose” of meditation training altering pain-related effects and its specific (or not) impact on activation of underlying pathways (including placebo physiology) has to be discussed. In addition there is the question whether meditation during or directly before pain application, or a short-term meditation training for novices as in our case, can easily be compared to mid-term or even long-term meditation practice regarding pain perception and its processing. Maybe some of the experiments conducted so far stressed more the cognitive appraisal of pain whereas others mainly tested emotion-related pathways (cf. Esch, 2014).

If, however, mindfulness in relation to pain was independent of placebo and related physiology in the brain, including opioid signaling, we would have to discuss more thoroughly the nature of pain reduction in its core sense, i.e., discerning primary pain inhibition from its secondary modulation, or distinguishing ascending, spinal or even peripheral processing of painful stimuli versus their descending, central or brain-derived (top–down) adjunct pathways; this differentiation would also include attention versus distraction models, or a distinction between perceived pain intensity on the one hand and perceived “bothersomeness” of pain on the other. Accordingly, subjective acceptance of pain, e.g., learning to “live with it,” would become a critical focus – as this is also part of the philosophical background of mindfulness (Hayes et al., 2004). Acceptance would thus be a psychological process by which attention toward pain, under certain conditions (e.g., low fear of pain, low catastrophizing, etc.), may even be beneficial. Clearly, pain is not just pain, instead, essentially an individual, subjective phenomenon – and an increased control of emotional reactions to pain would involve cognitive but also meta-cognitive pathways. Therefore, not only afferent pain signaling pathways would have to be examined more accurately in light of these speculations, but also secondary and tertiary responses to it, and the latter could be inhibited, one might speculate, through (experienced) meditation practice. In this scenario, afferent pain could be mentally suppressed, i.e., its appraisal and otherwise inhabited responses would be centrally altered or turned down, and consequently, pain “tolerance” would increase.

Following Williams (2010), failure to switch off emotion is due to the activation of mental representations of the present, past, and future that are created independently of external contingencies. Mindfulness training can thus be seen as one way to teach people to discriminate such “simulations” from objects and contingencies as they actually are. In addition, he argues, it might not only be the pain that is of importance here, but attending to the affected region of the body in an even-handed way: a problem in chronic pain would not only be the pain itself, but the ‘turning away’ from, the averting of attention from regions that give rise to painful sensations, either through deliberate distraction, or by thinking about the pain conceptually rather than experiencing the sensations directly. Some authors (cf. Craig, 2003) even regard pain itself an emotion, i.e., a distinct sensation *and* a motivation, calling it a specific emotion that reflects homeostatic behavioral drive, similar to temperature, itch, hunger, and thirst. Hence, pain *per se* would not be negative (or positive), yet a biological necessity for homeostasis, and pain modulation would primarily be considered emotion autoregulation. Farb et al. (2015) conclude that many of these processes can be understood through an emerging predictive coding model for mind-body integration. Their model, which describes the tension between expected and felt body sensation, parallels contemplative theories, and contemplative practices, such as meditation, may actually help to attenuate said interpretative biases. Given these speculations, one could possibly discriminate pain reduction in future experimental models incorporating meditation – or placebo – practices, with an involvement and activation of mechanisms that enhance the primary inhibition of pain, from pathways using an innate potential to control emotions. In other words: we would have to additionally look at individual pain-response-control potentials.

Taken together, in our experiments we saw the tendency that a brief mindfulness training resulted in a – relatively – *decreased* pain tolerance rather than an increased one. We suggest this could be due to the fact that mindfulness rather is a skill than having a directed ‘effect’ *per se* (e.g., on pain), especially when no direction of expected effects is implied (no specific outcomes predetermined, by default) and as such communicated to participants. We should keep in mind that mindfulness is not a method developed specifically for pain treatment. Moreover, this newly acquired skill of mindfulness could also lead to a more ‘sensitive’ perception of pain, that is, higher pain awareness over the course. Interestingly, a recent study by Sprenger et al. (2012) found that distraction reduces pain and that this effect is modulated by naloxone, pointing toward the fact that opioid neurotransmission is involved in attention-related pain perception. This effect might be opposite to (or: competing with) other pathways and neuronal mechanisms, or techniques, for pain modulation, such as meditation and mindfulness. We might have unintentionally activated different pathways for pain proceeding, that is, attention and awareness versus distraction, with a possibly pain-reducing effect of mindfulness being counteracted by a decrease of distraction (i.e., mindfulness would then, according to Sprenger et al. (2012), potentially antagonize a pain-reducing effect of distraction). Again, given the piloting character of our study, we can only speculate about said autoregulatory phenomena.

### Additional Limitations

Besides the question whether or not the chosen paradigm of our study was appropriate, we additionally discussed cultural effects and socioeconomic or regional cofounders on pain modulation, considering general – including structural – differences between Germany and other countries; such differences might have played a role here, since our study, to our knowledge, was the first one testing experimental pain tolerance in relation to a short mindfulness training in a German setting. One question, for example, would be whether having pain, given a certain health care and delivery system, is considered a signal to utilize and enter the system (having access to comprehensive care and coverage), versus a possible threat to individual function and dependent work issues and income options. Implications of pain might thus be quite different – related, also, to the question of social fallback systems, or pain as a threat to existential needs, including differences in (social) tolerability for pain.

Another limitation of our study might be that, although using standardized methodology and protocols, the ischemic pain stimulus wasn’t suitable for this kind of experiment. We tested pain stimulation via tourniquet method on ourselves, and we experienced an increasing unpleasantness and numbness rather than actual experience of ‘pain.’ Again, this raises the question whether pain perception might be a term susceptible to interpretation, implying social, but also to individual differences, and variance. Hence, the tendency of pain tolerance in our study to decrease – relatively – under naloxone (as compared to saline), could still point toward a more universal activation, or involvement, of opioidergic mechanisms in pain modulation. However, as described, we didn’t perform further analyses on this matter. Further research would thus be needed, and is strongly encouraged.

### Is Meditation a Placebo Intervention?

The main point for discussion, however, would be whether pain reduction or an increase of experimental pain tolerance through mindfulness meditation training, as it had been shown in other studies, but not in ours, appears to be a ‘placebo’ (rather learned) phenomenon, or, in fact, demonstrates an original (‘inherited’) potential of mindfulness *per se*. In other words: we carefully refrained from using the term ‘pain’ during the training, and also left it open to each participant going through the pain measurements to link the perception of pain – or its tolerance – to meditation. We thus tried to avoid active induction of expectations in this direction: this portion of a possible placebo response was purposefully prevented. Since the other portion, the conditioning part (supposedly involved here: elicited through the experimental procedures themselves), was the same for all participants, due to the study flow and the sequence of pain measurements, the only distinction at this point would be the training itself. Linked here is the discussion if meditation itself can be seen as a ‘medication’ (cf. Shapiro et al., 2006), say, whether meditation automatically and implicitly has specific, directed effects toward pain. Hence, meditation practice requires active involvement, and the participant’s decision, or effort, to learn the technique. This makes it almost the opposite of a typical medication, as this would usually be taken passively. We stress again that pain was not addressed in the meditation practice; neither was it part of the training to use the learned skill in such a way nor was its assumed connection made explicit during the experiments.

In another recent study by Zeidan et al. (2015) on meditation-based pain relief in comparison to placebo analgesia, a distinction could be made between the different pathways employed, with mindfulness meditation activating higher-order brain regions (e.g., orbitofrontal, anterior insular and cingulate cortices) and placebo analgesia deactivating sensory processing regions (e.g., secondary somatosensory cortex). Again, mindfulness meditation seems to correlate with the cognitive modulation of inner experience (e.g., pain), whereas placebo analgesia may primarily relate to the sensory regulation and inhibition of painful stimuli. However, the same study also demonstrated an activation of the dorsolateral prefrontal cortex in placebo analgesia, and the question remains unanswered whether mindfulness actually possesses an inherent pain-“specific” potential. Observed effects could also be due to a conditioning response and implications toward cognitive control of painful experiences, thereby proving an involvement of “unspecific” but now directed (conditioned) pathways for pain relief. In fact, meditation and placebo may very well overlap in this regard, and the existence of placebo analgesia (elicited via “analgesic” skin cream in this case), as a distinct neural entity, may not exclude the existence of a (“analgesic”) placebo-meditation-pathway.

### Future Study Designs

Clearly, further research seems to be necessary for a better understanding. For example, in a future study differentiating between non-intentional meditation training (as in our case) and another group where such intention is explicitly part of the training, we would suggest a three-armed study, with a passive control and an active training group (non-intentional), accompanied by a third active group that explicitly teaches mindfulness for pain control (as a “means for better tolerating pain”). In this latter group, participants would learn – and thus expect – to better cope and withstand pain, given the overall idea of acceptance, non-judging, and observing (as it is part of mindfulness training), even when facing a painful stimulus. With such a design, a possible overlap, as well as differences, between meditation and placebo outcomes, and physiology, could possibly be discerned more thoroughly.

However, this is a rather new area, and one could certainly not expect to understand the full picture of underlying mechanisms in only a few studies. Additional attempts are necessary. Also, long-term comparative cohort studies might be needed over the course to investigate the full potential of meditation techniques in relation to pain tolerability.

## Conclusion

A 5-day meditation training (approximately 460 min of mindfulness training/exposure) may not lead to increased pain tolerance.

Our results raise further questions: what part of assumed effects is related to expectation or conditioning? Which portion of earlier reported pain modulation through meditation is specific and actually meditation-related? Does meditation function as a ‘medication’? Should meditation be seen as a skill or potential, rather than producing discernable and predictable (objective, generally reproducible) outcomes?

We surmise that mindfulness may have the potential to reduce pain, but may not do so ‘automatically,’ as long as the use of this skill and expected outcomes are not trained simultaneously. When it occurs, this might also indicate intentionally expected or conditioned effects. Given a possible overlap between placebo studies and recent meditation studies on experimental pain, one could speculate a physiological interference between meditation and placebo phenomena. To better understand these questions, further studies are urgently needed.

## Ethics Statement

This study was carried out in accordance with the recommendations of the Internal Review Boards/Ethics Committees at Coburg University and Freiburg University Medical Center with written informed consent from all subjects. All subjects gave written informed consent in accordance with the Declaration of Helsinki. The protocol was approved by the Coburg University Ethics Committee, then confirmed by the corresponding ethics committee at Freiburg University Medical Center. Original ethics approval was obtained on December 20, 2012.

## Author Contributions

TE: grant application, study design, IRB procedures, methods, study execution, data management, data evaluation, manuscript preparation; JW: study execution, data management, data evaluation, manuscript preparation; VA: study design, methods, study execution, data management, data evaluation; HG: study execution, data management, data evaluation; RH: study design, methods, study execution, manuscript preparation; SS: grant application, study design, IRB procedures, methods, study execution, data management, data evaluation, manuscript preparation.

## Conflict of Interest Statement

The authors declare that the research was conducted in the absence of any commercial or financial relationships that could be construed as a potential conflict of interest.
